# Rise of Asian research in orthopaedic and sports medicine: a bibliometric analysis from 1996 to 2022

**DOI:** 10.1007/s00590-025-04294-5

**Published:** 2025-04-29

**Authors:** Sravya Teja Paleti, Srinivas B. S. Kambhampati, Abhishek Vaish, Raju Vaishya, Riccardo D’Ambrosi

**Affiliations:** 1 Alluri SitaRama Raju Academy of Medical Sciences, Andhra Pradesh, India; 2 Sri Dhaatri Orthopaedic, Maternity & Gynaecology Center, Andhra Pradesh, India; 3 Indraprastha Apollo Hospitals, New Delhi, India; 4 Istituto Ortopedico Galeazzi, Milan, Italy; 5 Dipartimento di Scienze Biomediche per la Salute, University of Milan, Milan, Italy

**Keywords:** Research, Asia, Orthopaedics, Sports medicine, Bibliometrics

## Abstract

**Objectives:**

This study examines the growth and impact of orthopaedic and sports medicine (OSM) publications across 30 Asian countries from 1996 to 2022 using a bibliometric (scientometric) approach. Despite Asia’s rising academic achievements, prior studies have not comprehensively mapped publication trends in this field across the region. This analysis aims to perform bibliometric analysis in OSM research in the Asian Countries.

**Methods:**

Publication data were sourced from the SCImago Journal & Country Rank portal, derived from the SCOPUS database, covering the period from 1996 to 2022, with updates available until April 2023. The analysis focused on the top Asian countries and included key indicators such as H-index and total citations to assess research impact.

**Results:**

The study identified a substantial rise in OSM publications from Asia, with total output increasing 14.27-fold—compared to a 5.54-fold increase globally. Between 1996 and 2022, 111,342 OSM publications originated from Asian countries, out of 666,847 globally. However, citation counts for Asian research declined from 26,263 in 1996 to 6020 in 2022, likely reflecting the time-lag effect in citation accumulation for recent publications. Possible contributing factors are discussed.

**Conclusion:**

This study highlights a remarkable surge in orthopaedic and sports medicine publications from Asia, surpassing global growth trends. While citation metrics appear lower in recent years—likely due to the recency of publications—the overall trend suggests a strong and growing research in Asia. China and Japan lead in output and impact, respectively, while India’s rapid rise reflects increasing academic potential. However, enhancing research quality and visibility and reducing self-citation are essential to elevate global impact. Countries like Hong Kong, Singapore, and Sri Lanka demonstrate high citation efficiency. With optimal collaboration and strategic investment, Asian countries are well positioned to play a leading role in global orthopaedic research.

## Introduction

In the evolving landscape of global research and publications, analysing the trajectory of academic output from various countries offers critical insights into their scientific progress and impact. China was the first nation to start producing paper by hand around 2000 years ago, and Asia, as a continent, has a rich history of medicine and medical systems dating back to hundreds of years Before Christ. Systems like Ayurveda from India and traditional Chinese medicine were among the first developed systems. However, modern orthopaedic publications emerged only in the nineteenth and twentieth centuries. With the merging of numerous technologies into orthopaedics and developing Asian countries in the software industries, the numbers are only expected to increase significantly. Asian orthopaedic publications are increasingly recognized in the global medical community, with many researchers collaborating internationally. Regional challenges are unique in Asian countries, and these are brought out in these publications.

Asia has a very diverse social, linguistic, demographic, cultural, and economic structure between its countries. This adds to the challenges faced in the production of English speaking literature. Clubbed with this, different countries are developing at different paces. While China and Japan are among the most developed countries, Bangladesh and Sri Lanka are lagging behind and this produces its own challenges in creating the optimal conditions for sporting activities and research in this field. There is wide variation in the adaptation of research methodologies as a result. A bibliometric study at this time would highlight the gap in both the quantity and the quality of the literature in the field of sports medicine between the different countries.

In a Chinese study in 2016, the USA contributed the most significant proportion (31 190 (24.20%)), followed by the UK (6703 (5.20%)), Japan (5718 (4.41%)), Germany (4701 (3.66%)), and Mainland China (MC) (3389 (2.63%)). Articles from China accounted for the least number compared to other leading nations in this paper [1]. This trend has improved significantly in the last two decades [2]. Chinese universities have been playing an increasingly important role in global health research, as assessed by peer-reviewed publications from 2014 to 2020 [3]. China’s contribution to biomedical research blossomed after 1990 and especially after 2000 [4]. With increasing publications from India and other Asian nations, we wanted to look at the publication trends among Asian countries in the speciality of orthopaedics and sports medicine (OSM), using Scopus data from the SCImago website (SCImago). In addition, several essential publication metrics like citations, self-citations, citations per document (CPD), and H-index were evaluated, as they provide a more nuanced understanding of the research landscape, revealing not just the quantity but also the quality and influence of the research outputs from these countries. This bibliometric review will also be helpful for strategic planning, collaborations, and policy-making in orthopaedics and sports medicine.

## Methods

We used the SCOPUS data from the SCImago Journal & Country Rank website. It provides free access to several publication metrics from 1996 to 2022 and was updated in April 2023. Our search strategy was as follows:

(A) For All Country Regions: SCImago website > > Country ranking > > All Subject Areas > > Orthopaedics & Sports Medicine > > All Regions > > Year (1996–2022 and 2022).
(B) For Asia: SCImago website > > Country ranking > > All Subject Areas > > Orthopaedics & Sports Medicine > > Asiatic region > > Year (1996–2022 and 2022).

The data of these respective fields were downloaded into Excel sheets for analysis for several important publication metrics like total documents, citations, self-citations, citations per document, and H-index. The country rankings of Asia in OSM were evaluated and compared from 1996 to 2022 (27 years).

We looked into the trends of publications and compared between all countries and Asian countries as well as between Asian countries. We also looked into the productivity and citation counts and the research impact of the Asian countries and compared the number of publications, citations, and H-index between the periods of 1996 and 2022 for which the data were collected.

T statistic, *p* value, and effect size (Cohen’s d) were calculated between the period of 1996 and 2022 for document counts for all countries, Asian countries, and their citation counts and the results analysed.

## Results

**Fig. 1:**
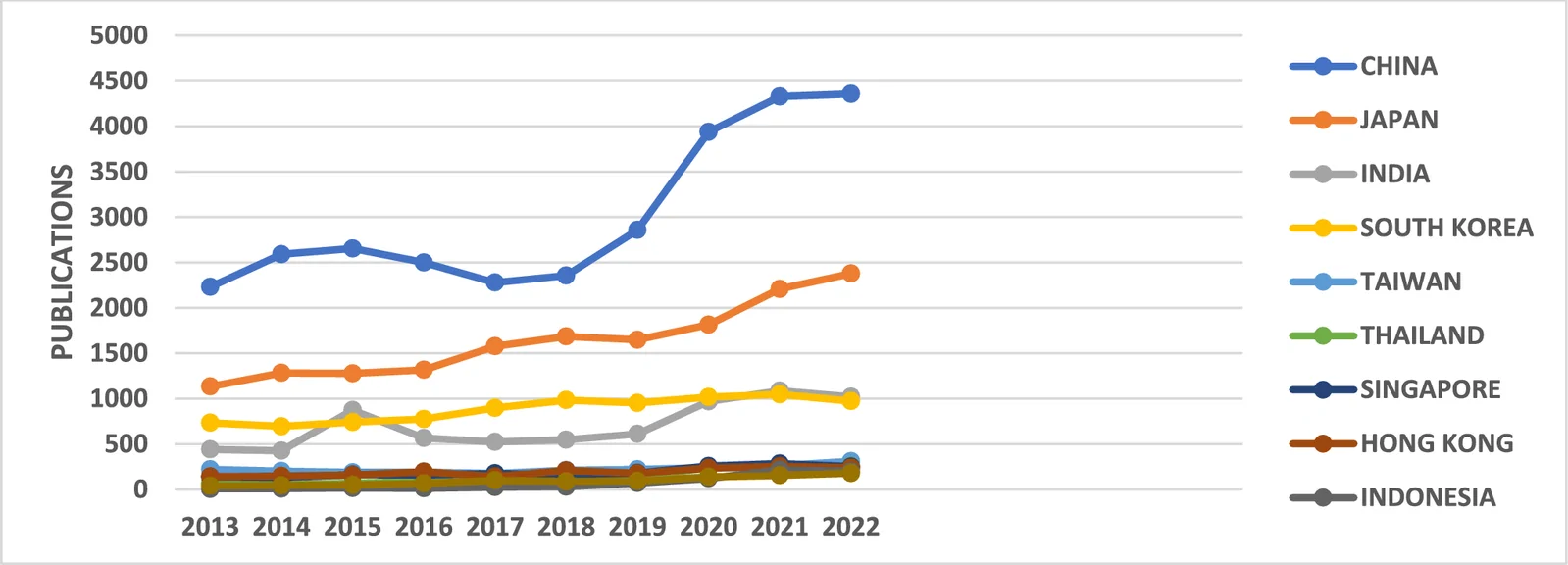
Total number of publications by Asian countries

In the study period from 2013 to 2022, there was a massive increase in the number of publications by China from 2231 publications in 2013 to 4357 in 2022, followed by Japan and India (Fig. 1). Japan shows a high output despite a more stable or mature growth rate. Other significant contributors include South Korea, Taiwan, Iran, Turkey, Pakistan, Indonesia, and Malaysia.

When the trends of publications were observed, China published a constantly high number of articles, but the maximum increase in number was in 2020. Indonesia has increased the number of publications exponentially from 2013 to 2022 (Table 1).

**Table 1:** Year-wise publication counts from 2013 to 2022 across major Asian countries

Year	China	Japan	India	South Korea	Taiwan	Thailand	Singapore	Hong Kong	Indonesia	Malaysia
2013	2231	1134	441	735	222	70	140	142	5	41
2014	2590	1284	427	696	203	58	132	149	9	45
2015	2653	1279	874	742	189	72	164	159	16	51
2016	2499	1318	567	776	191	90	160	196	13	69
2017	2279	1577	524	898	176	176	177	142	25	101
2018	2356	1685	548	984	213	123	157	210	31	90
2019	2857	1648	612	953	222	147	181	178	71	95
2020	3938	1815	970	1018	242	235	259	235	122	139
2021	4328	2208	1087	1047	264	260	286	254	217	156
2022	4357	2378	1020	974	310	258	252	240	200	180
Total	30,088	16,326	7070	8823	2232	1489	1908	1905	709	967
Average	3008.8	1632.6	707	882.3	223.2	148.9	190.8	190.5	70.9	96.7

Table 1 presents the annual research output (number of documents published) from 10 Asian countries over a 10-year period. From the presented data, one can see that China leads with a total of 30,088 publications, averaging over 3000 per year, and showing a consistent increase over the decade. Japan follows with India and South Korea ranking third and fourth. 2020–2022 marks the peak period for most countries, reflecting an acceleration in research output during or after the COVID-19 pandemic era.

### Trends of publications: All countries vs. Asian countries

From 1996 to 2022, 666847 were publications listed for OSM for all countries and 111342 for Asian countries, accounting for a share of 16.66%. In 1996, the contribution of Asian countries to global OSM publications was only 7.72% (726/9402), which rose to 19.88% (10363/52120) in 2022. There was an overall growth of 14.27 times (10363/726) of the Asian publications in OSM from 1996 to 2022, compared to 5.54 times (52120/9402) of all countries.

### Trends of publications among Asian countries

Out of 30 participating Asian countries in OSM publications from 1996 to 2022, the top five contributed 90.25% (100,485/111342) of the total publications. These top five countries were China (*n* = 43,866), Japan (*n* = 30,307), South Korea (*n* = 12,669), India (*n* = 9331), and Taiwan (*n* = 4312).

The year-on-year growth was maximum for Indonesia (Table 2); however, the number of its overall publications was low compared to other Asian countries. The standard deviation (SD) for Indonesia was more than the average, indicating that a median better represented the central measure in this case, which is 28.

**Table 2:** Growth of publications for each country in the last decade

Year	China	Japan	India	South Korea	Taiwan	Thailand	Singapore	Hong Kong	Indonesia	Malaysia
2013–2014	16.09	13.23	− 3.17	− 5.31	− 8.56	− 17.14	− 5.71	4.93	80.00	9.76
2014–2015	2.43	− 0.39	104.68	6.61	− 6.90	24.14	24.24	6.71	77.78	13.33
2015–2016	− 5.80	3.05	− 35.13	4.58	1.06	25.00	− 2.44	23.27	− 18.75	35.29
2016–2017	− 8.80	19.65	− 7.58	15.72	− 7.85	95.56	10.63	− 27.55	92.31	46.38
2017–2018	3.38	6.85	4.58	9.58	21.02	− 30.11	− 11.30	47.89	24.00	− 10.89
2018–2019	21.26	− 2.20	11.68	− 3.15	4.23	19.51	15.29	− 15.24	129.03	5.56
2019–2020	37.84	10.13	58.50	6.82	9.01	59.86	43.09	32.02	71.83	46.32
2020–2021	9.90	21.65	12.06	2.85	9.09	10.64	10.42	8.09	77.87	12.23
2021–2022	0.67	7.70	− 6.16	− 6.97	17.42	− 0.77	− 11.89	− 5.51	− 7.83	15.38

### Research productivity

**Fig. 2:**
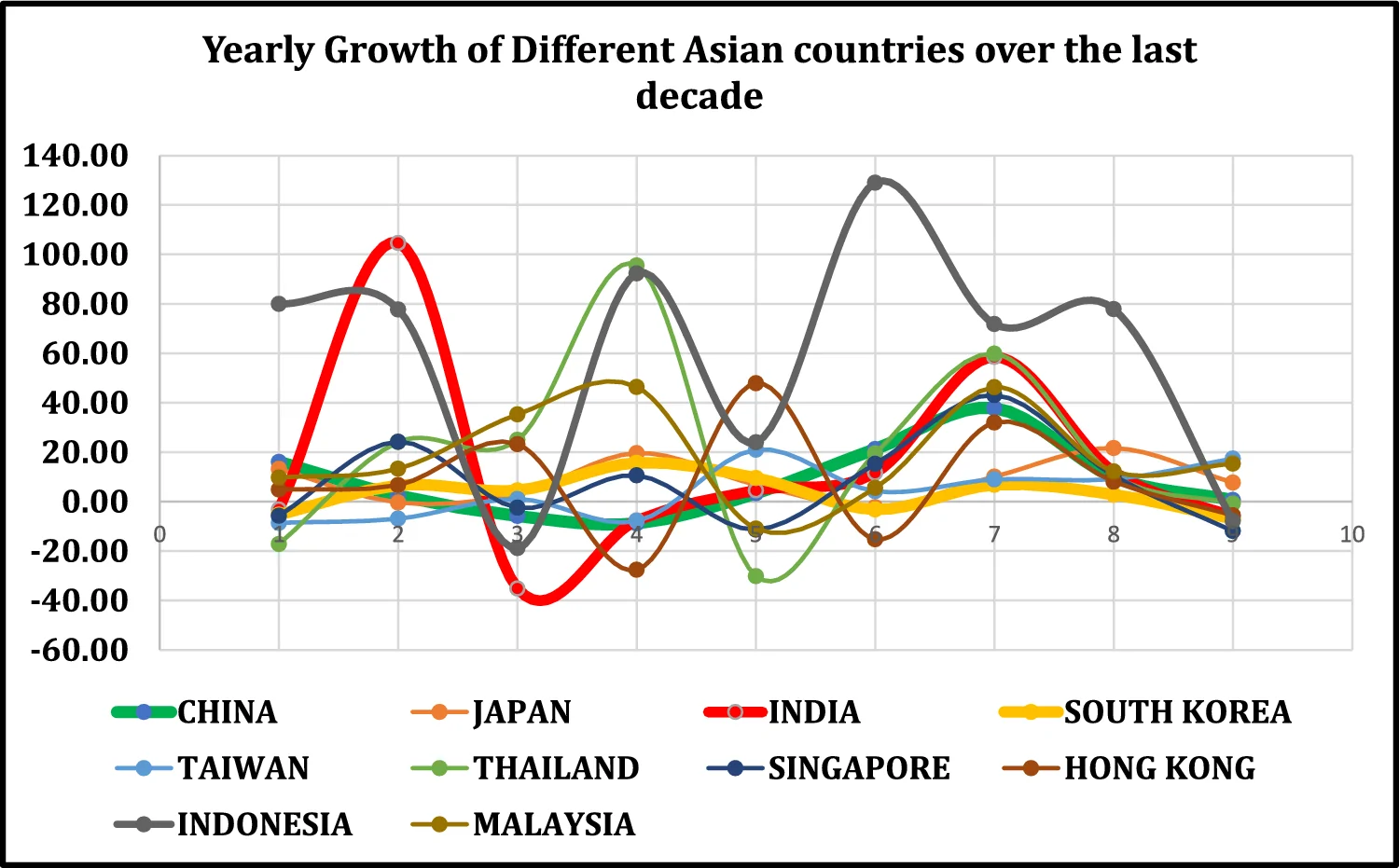
Annual growth rates of publications among Asian countries

There was a consistent growth year on year for Indonesia (Table 2), but with low numbers (starting at five and ending at 709) compared to the top three countries with the same numbers given in parentheses—China (2231, 4357), Japan (1134, 2378), and South Korea (735, 8823). India and Singapore had the highest number of years of negative growth on four occasions, while Malaysia had the lowest number of years of negative growth with one. The magnitude of negative growth was highest for India at − 35.13, while Indonesia had the maximum growth magnitude among all countries at 129 in 2018–2019 (Fig. 2).

Figure 2 displays the year-on-year growth in scholarly publication output from various Asian countries. Indonesia is prominent with the highest annual growth rate, despite having a relatively smaller total output. Countries such as China, India, and South Korea also show consistently strong growth, reflecting increasing investments in R&D and academic infrastructure. Japan, in contrast, shows lower growth rates, possibly due to a mature and already saturated research system. The standard deviation exceeds the mean for Indonesia, indicating high year-to-year variability, possibly due to changing research funding, indexing improvements, or policy shifts.

### Research impact

**Fig. 3:**
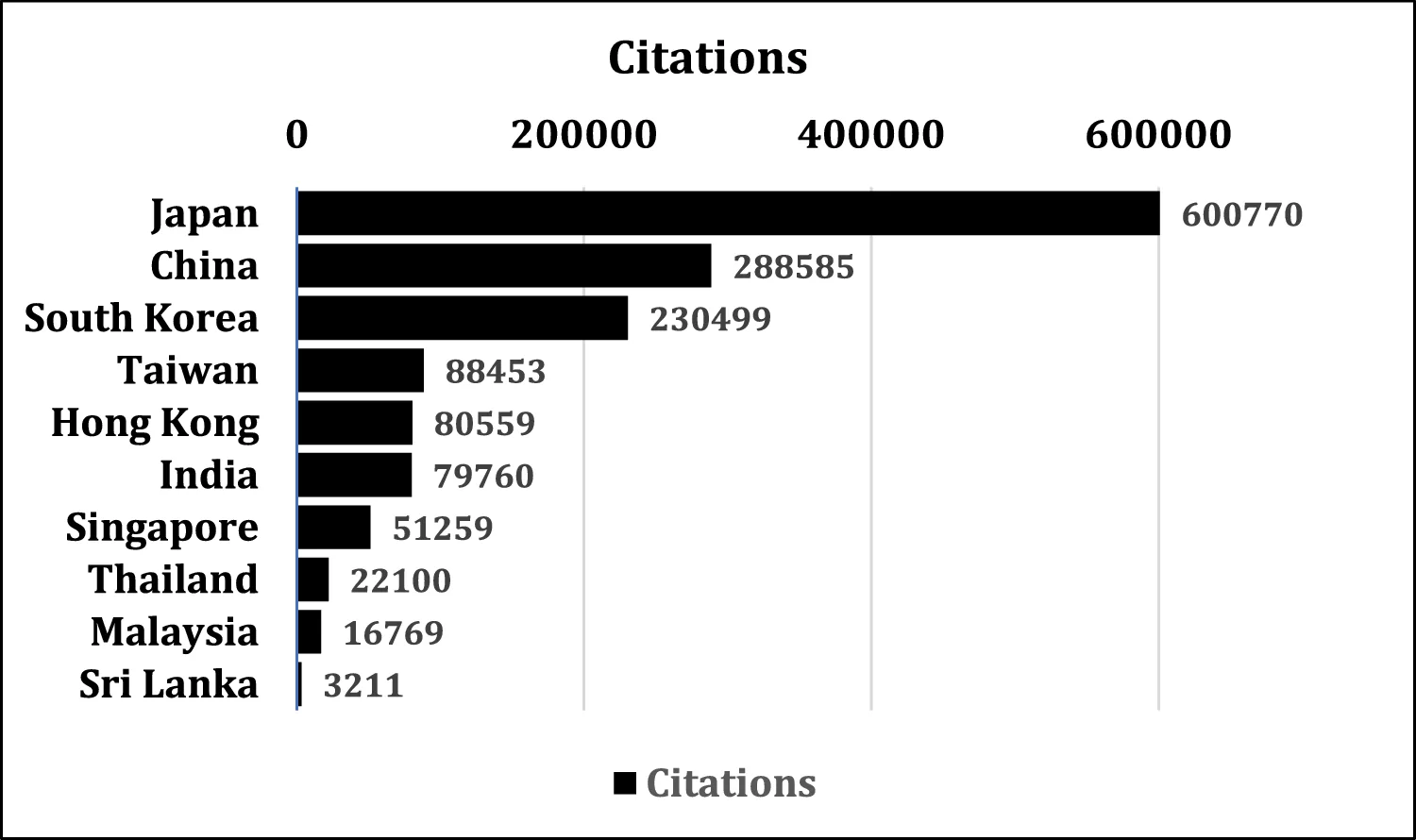
Total citation counts of the top 10 most-cited Asian countries

China has a high research impact, as evidenced by the high number of publications and citations. Japan has a high H-index, indicating a significant impact and longevity of research. India and other South Asian countries show a growing trend of publications. Taiwan and South Korea have many CPDs, indicating the high quality or influence of their publications (Fig. 3).

Japan leads with the highest total citations of 600,770 (Fig. 3), indicating strong historical research influence and globally recognized publications. China, while second in citations, lags significantly behind Japan, with less than half of Japan’s total citation count. Other countries in the top 10 include India, South Korea, Taiwan, Singapore, Hong Kong, and Turkey. The percentage of self-citations (Number of self-citations × 100/Total citations) was highest for China at 40%, followed by Japan at 22.2%. Although India is the fourth most published, it is only the sixth most-cited country.

**Fig. 4:**
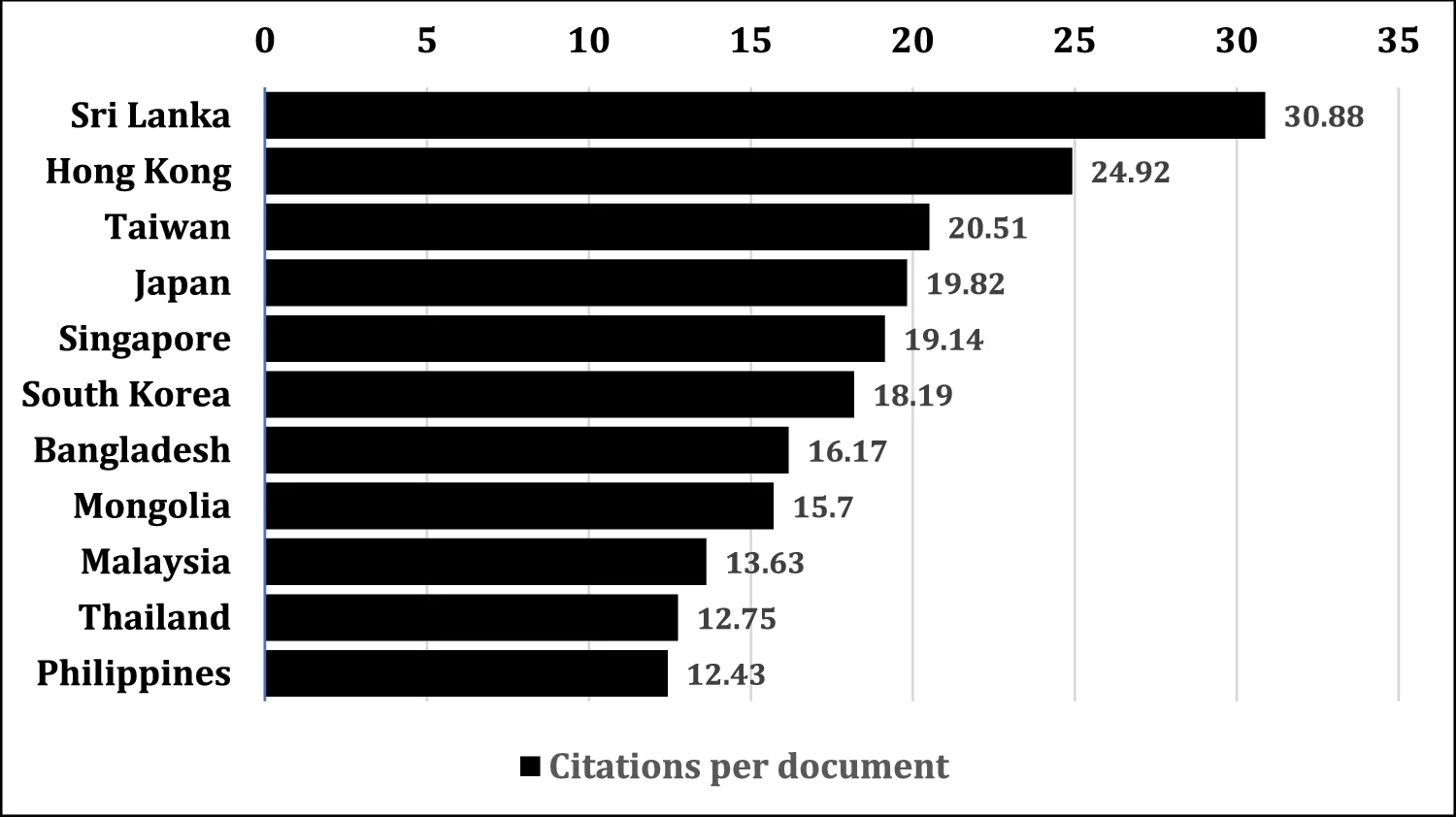
Citations per document (CPD) by country—ranked in descending order

Figure 4 shows countries such as Hong Kong, Singapore, or Sri Lanka are likely among the top performers, achieving higher CPD values. Countries with high publication volumes, like China or India, may appear lower on the CPD scale despite large outputs. This ranking indicates quality and influence per article, rather than sheer volume. China had the second highest number of citations and the most publications, and it stood at 22nd in CPD among the 30 studied countries (Fig. 4). It indicates that the impact of publications from China is limited. The country with the most CPD was Sri Lanka, with a value of 30.88, followed by Hong Kong at 24.92.

**Fig. 5:**
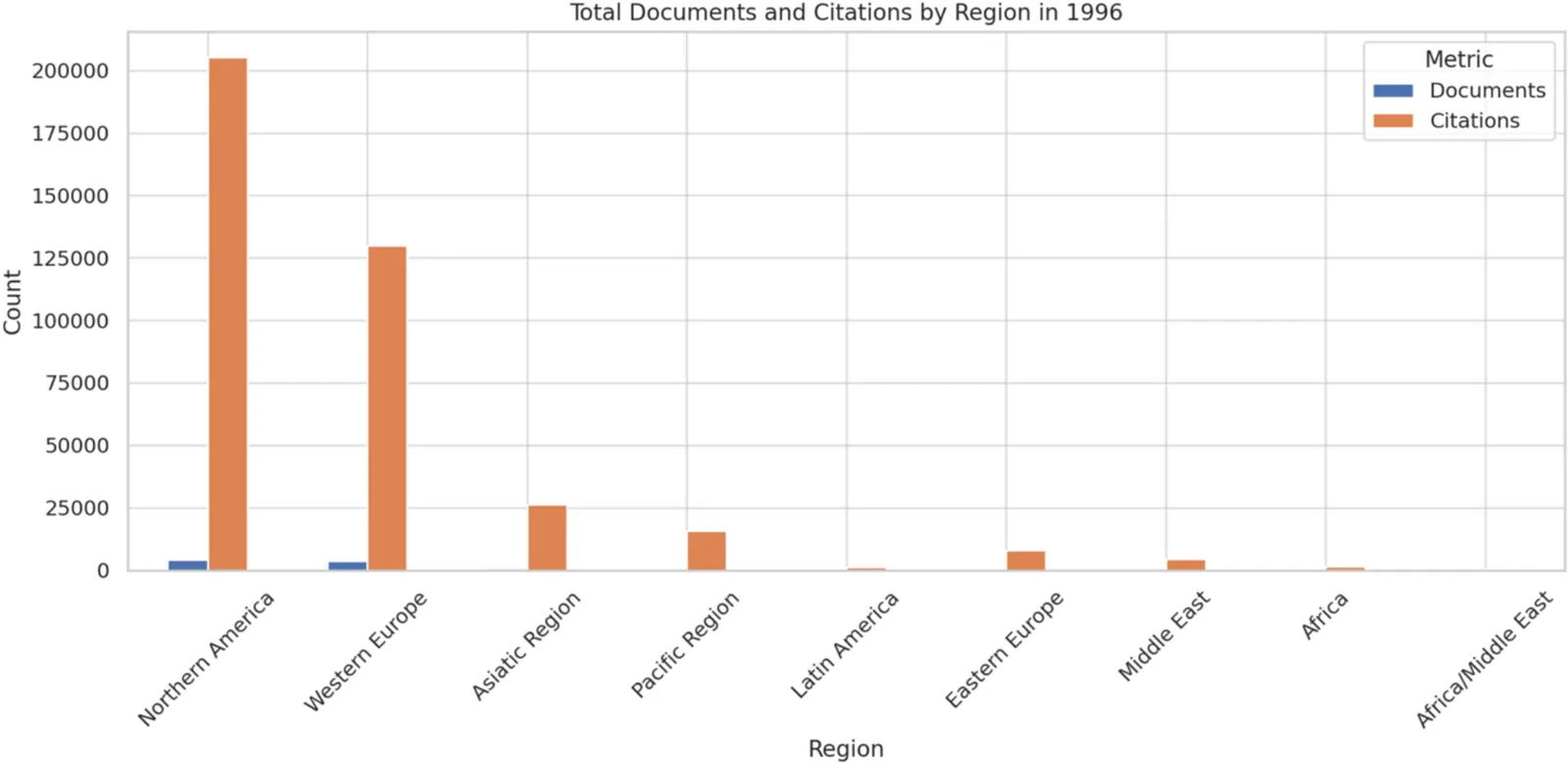
Comparison of average documents, citations, citations per document, and H-index between 1996 and 2022 for all countries and Asian countries. While publication volume has increased substantially over time, average citations, citations per document, and H-index values are lower in 2022, likely due to the recency of publications and insufficient time for citation accumulation

There has been a substantial increase in the number of published documents, especially from Asian countries, which surpassed the global average in 2022 (Fig. 5). This indicates explosive growth in research output across Asia over the past two decades. Despite the increase in publication volume, the average number of citations per country has dropped significantly in 2022 compared to 1996. This likely indicates the short citation window for newer publications, which have had limited time to accumulate citations. There is a dramatic reduction in citations per document over time. This is again due to the recency of publications in 2022, meaning papers have not had enough time to be cited. Interestingly, Asian countries slightly outperformed the global average in this metric, possibly due to improved visibility or quality of selected publications. The H-index has decreased for both groups. While this may seem counterintuitive, it reflects the fact that the H-index is citation-dependent and requires time to grow. Since 2022 publications are recent, they have not yet had the opportunity to generate high-impact citations groups of documents.

These comparisons reveal substantial changes in publication outputs and citation patterns over 26 years, globally and in Asian countries. It is essential to consider potential factors influencing these changes, including the expansion of academic publishing, evolving research landscapes, and changes in citation practices.

#### Key findings from documents and citations (Fig. 6a, b)

**Fig. 6:**
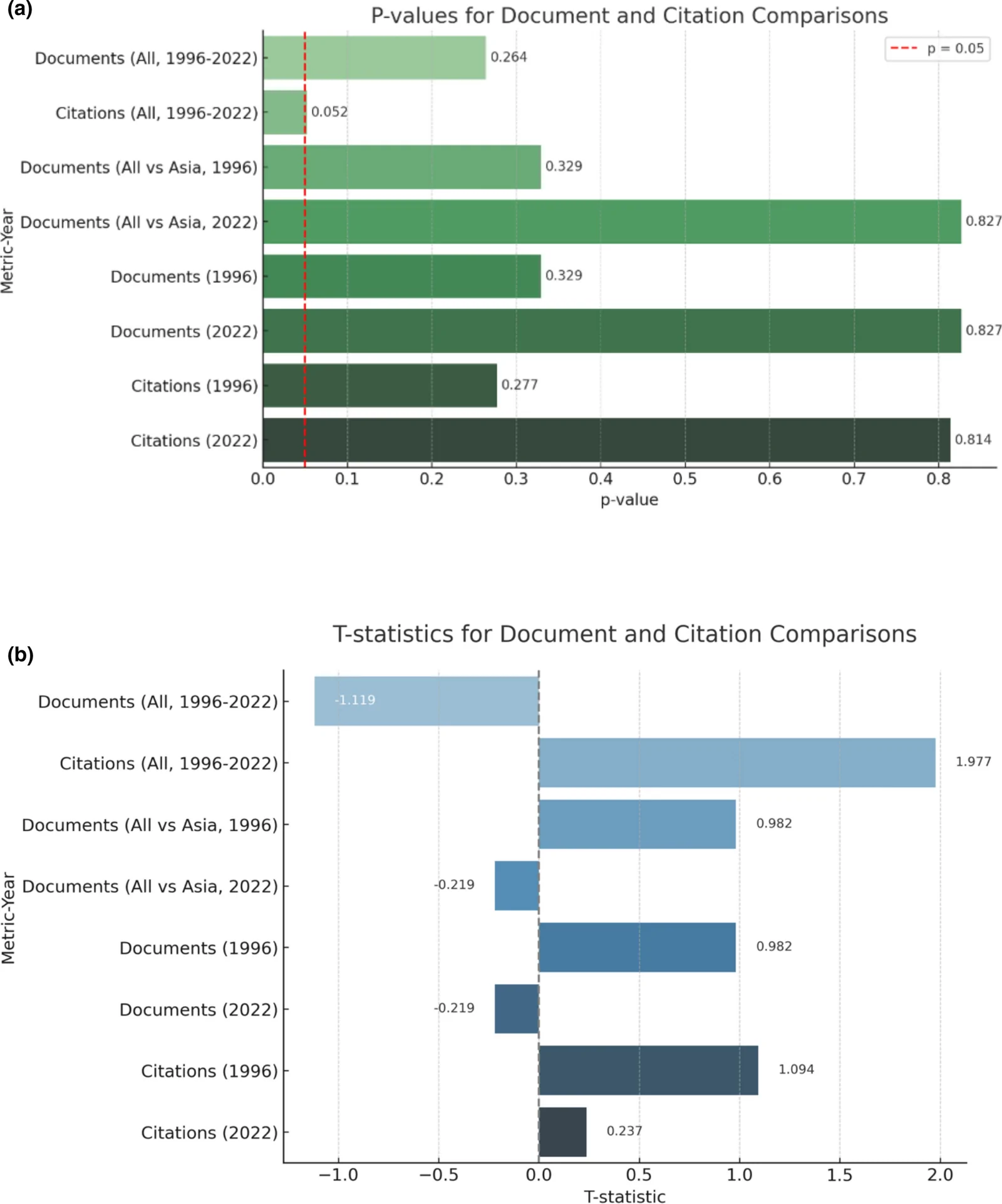
**a** *P* values for comparisons of document and citation metrics across years (1996 vs 2022) and regions (All countries vs Asia) The red dashed line at *p* = 0.05 indicates the threshold for statistical significance. None of the comparisons reach significance, though citation growth from 1996 to 2022 approaches it (*p* = 0.052). **b** T statistic for document and citation comparisons across time and regions. The chart visualizes statistical differences in publication volume and citation counts between years (1996 vs 2022) and between all countries and Asian countries. Positive values indicate higher means in the second group compared, while negative values indicate higher means in the first. The largest effect was observed for citation count comparison over time (*T* = 1.977)

##### Citation counts (All, 1996–2022)

With a T statistic = 1.977, *p* = 0.052, this is close to statistical significance. It suggests that there was a noticeable increase in citation counts between 1996 and 2022 for all countries combined. Although just above the *p* = 0.05 threshold, it approaches significance, implying a potential trend worth noting (especially in exploratory research).

##### Document counts (All, 1996–2022)

With a *T* statistic = −1.119, *p* = 0.264, The negative *T*-value indicates a decrease in average document count from 1996 to 2022. However, the *p* value is not significant, suggesting the difference could be due to chance.

##### Documents (All vs Asia, 1996) and documents (1996)

With a *T* statistic** = **0.982, *p* = 0.329, these show a mild difference in publication output between Asian and non-Asian countries in 1996. Again, the p values are well above 0.05, indicating non-significance.

##### Documents (All vs Asia, 2022) and documents (2022)

With a *T* statistic = −0.219, *p* = 0.827, this shows virtually no difference between Asian and non-Asian countries in 2022 in terms of document output. The very high *p* value confirms no statistically significant difference.

##### Citations (1996)

With a *T* statistic = 1.094, *p* = 0.277, it suggests a moderate difference in citation counts across groups in 1996. However, the p value indicates non-significance.

##### Citations (2022)

With a *T* statistic = 0.237, *p* = 0.814, this is the smallest effect, both statistically and practically.

It shows minimal difference in citation performance between regions in 2022.

There may be a genuine increase in overall citation activity across the years, though more data would be needed to confirm it definitively. There is no statistically meaningful change in total document counts across this time period. By 2022, the publication output between Asian and non-Asian countries had largely evened out. The citation behaviour appears similar across regions in recent years, perhaps due to globalization of research dissemination. This analysis indicates that while there is a noticeable difference in citation counts between the two years, the difference is not statistically significant with a high confidence level. However, the near-significance might suggest a trend worthy of a further investigation, perhaps with more detailed data or additional years for analysis.

In all cases, the p values are higher than the conventional significance level of 0.05. It indicates no statistically significant differences in the mean number of documents or citations between all countries and Asian countries for both 1996 and 2022.

### Comparing document numbers and citations among countries in 1996

**Fig. 7:**
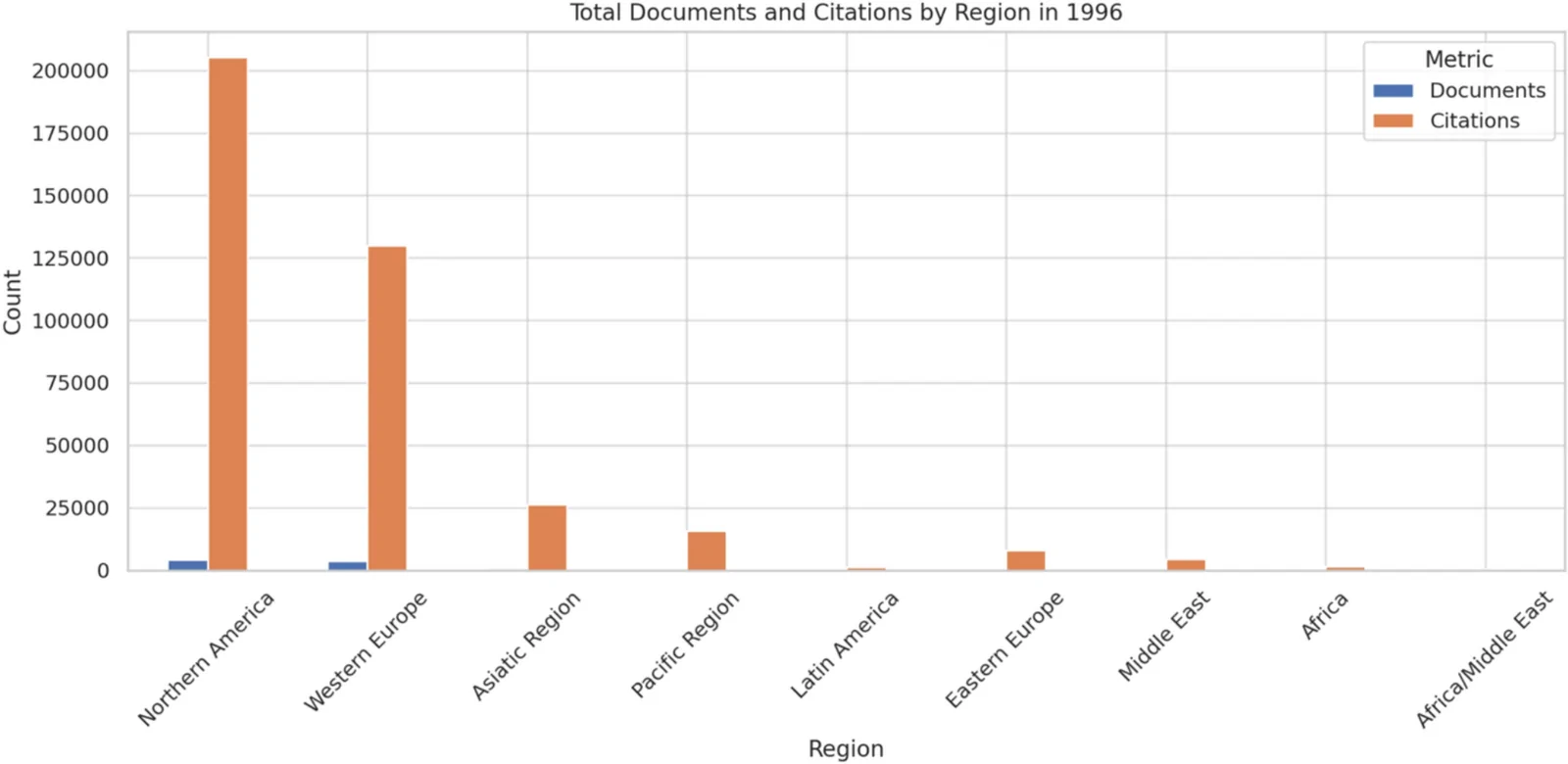
Total documents and citations by country in 1996

The comparative analysis for the year 1996, focusing on documents and citations among different regions, yielded the following insights (Fig. 7). Northern America had the highest publication output, with 4259 documents, receiving 2,05,365 citations. Western Europe followed with 3611 documents and 1,29,870 citations. The Asiatic Region, which includes Asian countries, had a total of 726 documents and 26,263 citations. Other regions like the Pacific, Latin America, Eastern Europe, and the Middle East had significantly lower outputs regarding documents and citations.

### Comparing document numbers and citations among countries in 2022 (Fig. 8)

**Fig. 8:**
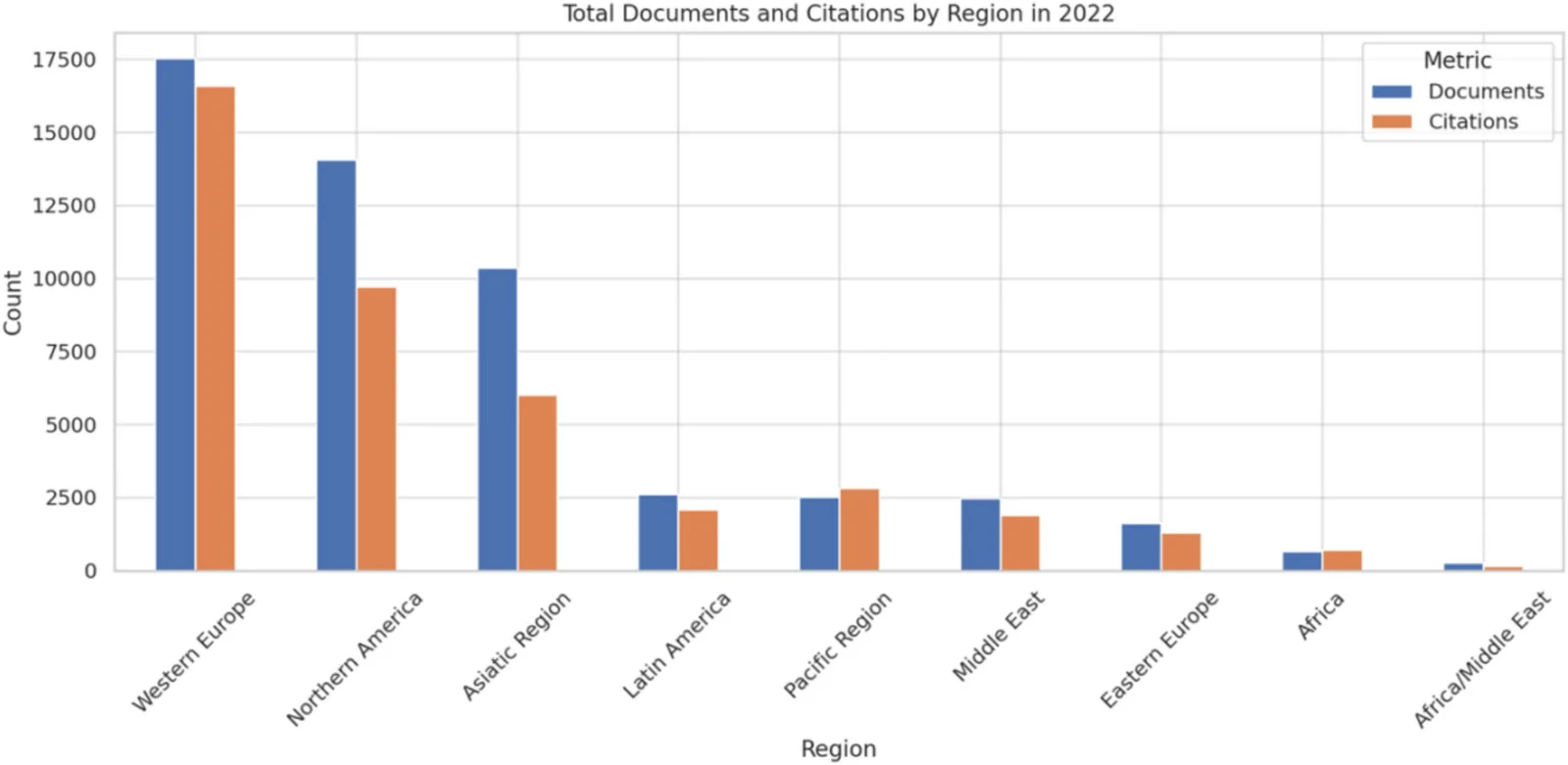
Total documents and citations in 2022

Western Europe led in publication output with 17,536 documents and received 16,598 citations. Northern America had 14,065 documents and 9711 citations, indicating a shift in the leading position compared to 1996. The Asiatic Region showed significant growth with 10,363 documents and 6020 citations, highlighting increased research output compared to 1996. Regions like Latin America, the Pacific Region, the Middle East, and Eastern Europe also showed increased outputs but remained lower than the leading regions. Africa and the combined region of Africa/Middle East continued to have the most miniature publication output and received citations, although there was a noticeable increase compared to 1996.

Figure 7 provides a historical snapshot of global research distribution and influence nearly three decades ago. The number of documents and citations was relatively low across most countries compared to present-day metrics. A few countries dominated both document numbers and citations—e.g. the USA, Japan, and possibly early contributors from Europe or Asia (e.g. China, India). The gap between documents and citations varied, indicating differences in research influence, visibility, and collaboration practices at the time.

This comparison indicates a significant shift in the global research landscape, with Western Europe taking the lead in publication outputs by 2022 and notable growth in the Asiatic Region (Fig. 8).

### Key observations on output from Asian countries

**Fig. 9:**
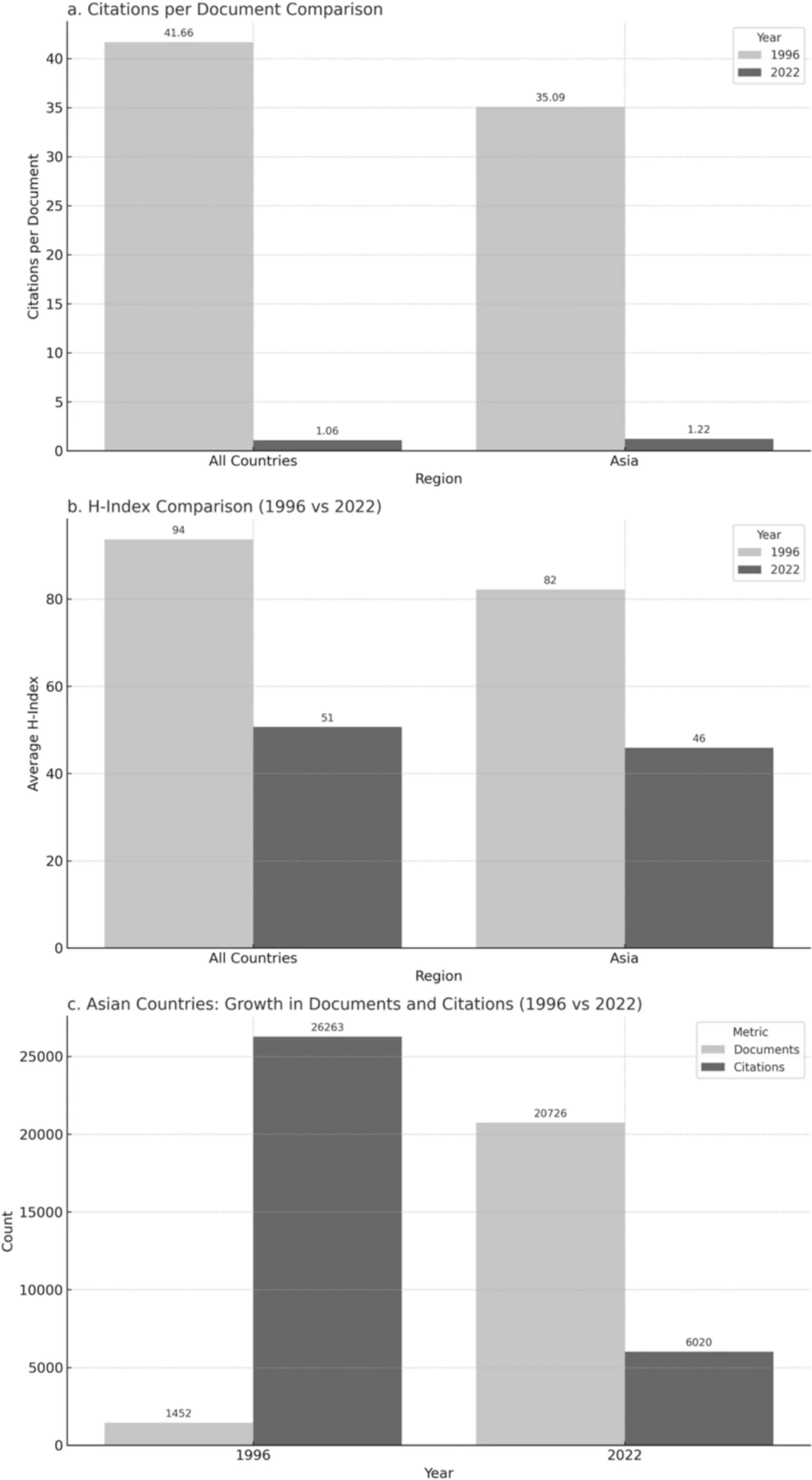
Comparative trends in citations per document **a**, H-index **b**, and research output in Asian countries between 1996 and 2022 **c**

As seen from Fig. 9a, citations per document have decreased significantly across all regions from 1996 to 2022, though Asia showed a slightly higher ratio than the global average in 2022. In Fig. 9b, H-index values have also declined over time, reflecting the limited opportunity for recent papers to accumulate citations. In Fig. 9c, despite these drops in impact metrics, total research output (documents) and citation volume in Asian countries have increased substantially from 1996 to 2022.

The data indicate substantial increases in the number of documents from 1996 to 2022 for both all countries and Asian countries but a decrease in average citations and H-index. It might reflect a broader trend in academic publishing where the volume of publications has increased. However, the average impact per publication (as indicated by citations and the H-index) has decreased.

This comprehensive analysis provides a clear picture of the evolution of research outputs and impacts across different regions, specifically in Asian countries. The significant decrease in citations per document could be attributed to various factors, including changes in research and publication practices, data collection methods, or the rapid increase in publications diluting the average citation count.

**Fig. 10:**
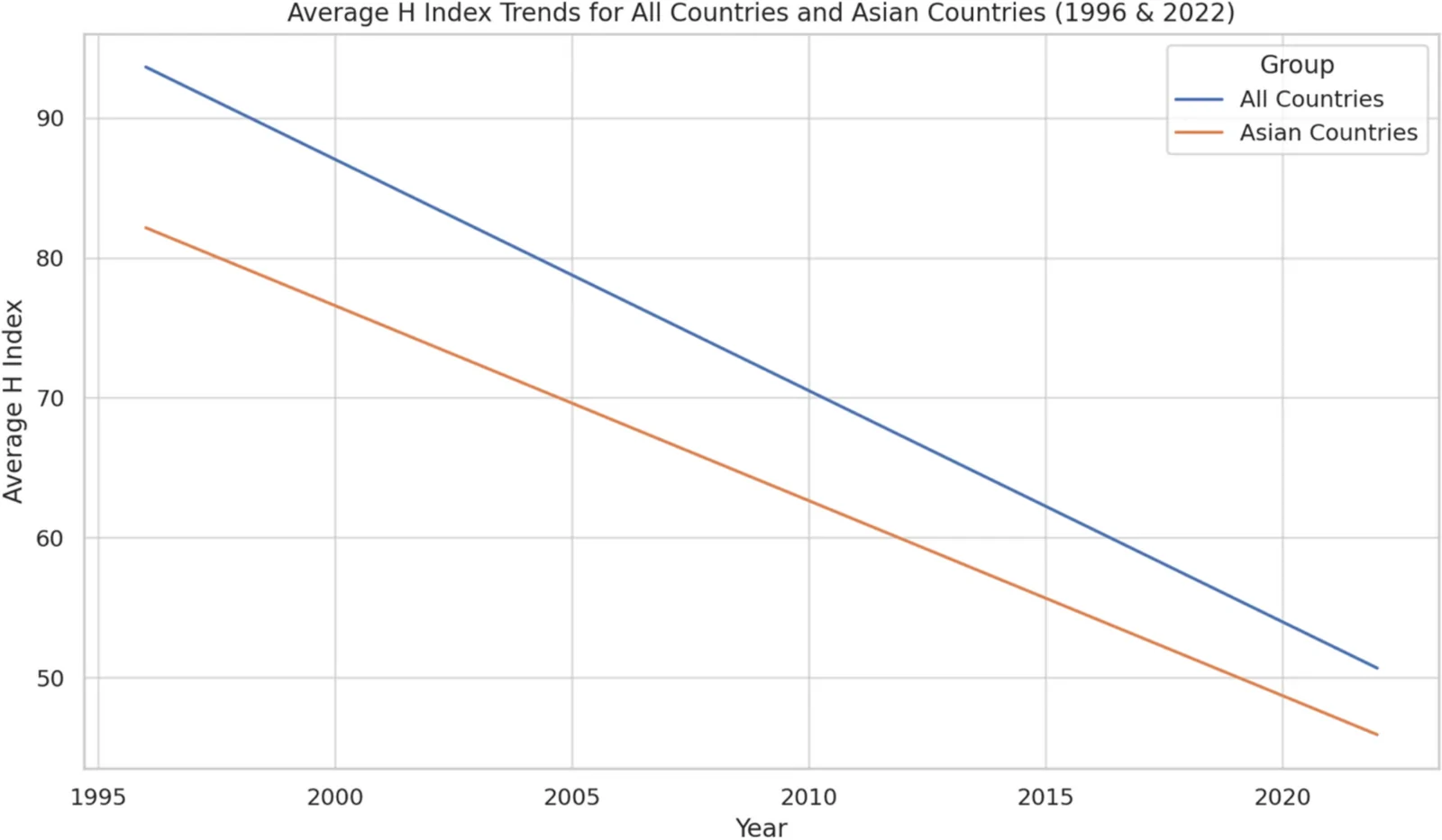
Comparison of H-index values of all countries

The line graph (Fig. 10) displays the variation in H-index across different countries, offering a comparative view of each nation’s cumulative research impact. The H-index captures both the quantity and citation strength of scholarly publications. Countries such as the USA, China, and Japan likely sit at the higher end of the H-index curve (Table 2 and Fig. 10), reflecting their dominance in global academic output. The gap between countries in H-index can highlight disparities in: research funding and infrastructure, international collaborations, and access to high-impact journals.

**Fig. 11:**
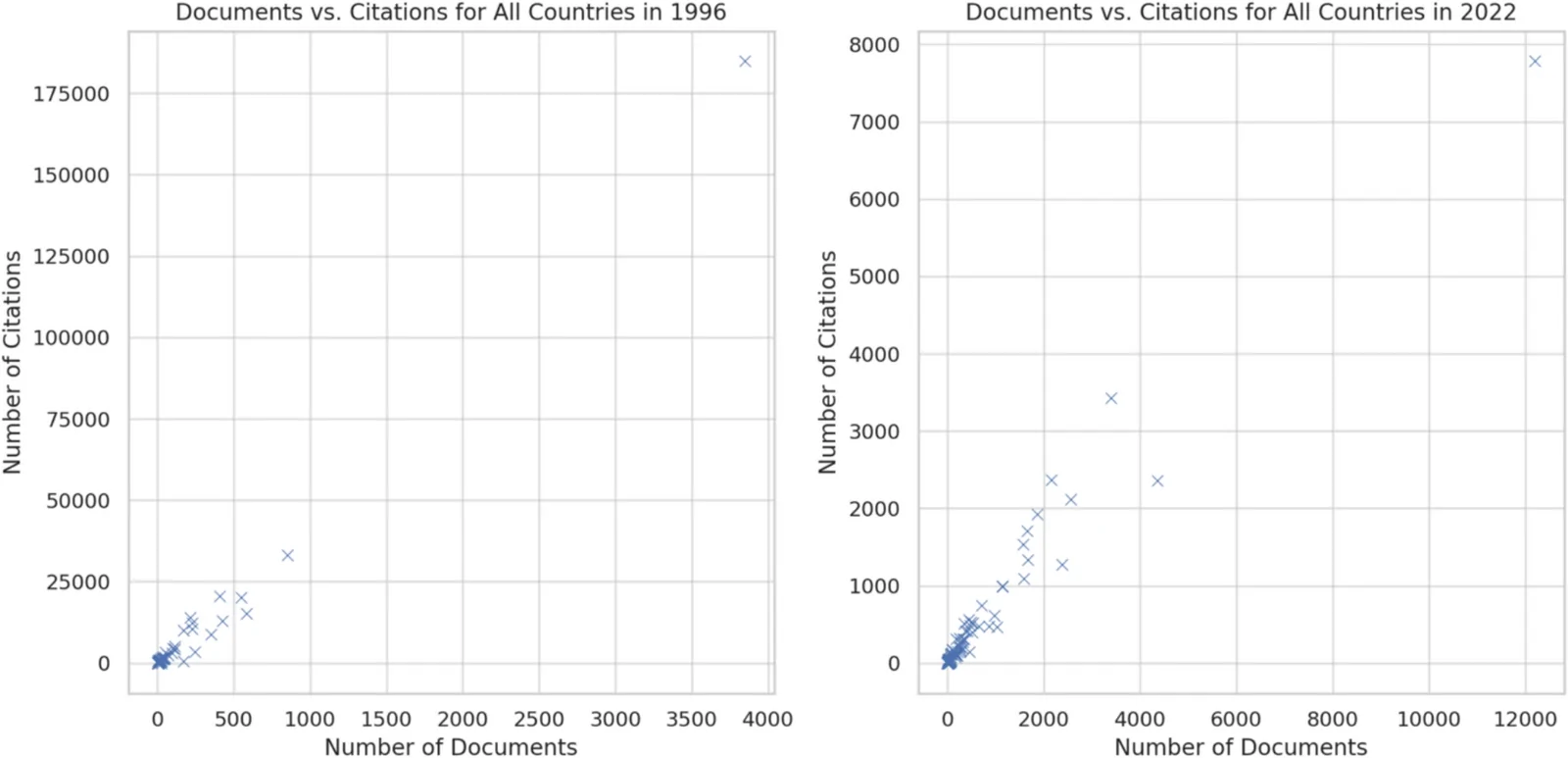
Correlation between the number of documents and number of citations across countries

The scatter plot (Fig. 11) illustrates the relationship between research output (number of documents) and research impact (number of citations) for various countries. Each point represents a country, with its position determined by its document count and citation count. A generally positive trend is observed, where countries with higher publication volumes tend to accumulate more citations.

## Discussion

This study confirms that China has produced the highest number of publications related to OSM, while Japan has garnered the most citations—600,770 in total—followed by China with 288,585 citations, which is less than half of Japan’s count. Since the economic reform of 1978, China’s medical field has made remarkable progress over the past four decades. In recent years, the continuous expansion of medical research teams and advancements in laboratory equipment and methodologies have enabled China to achieve several influential and internationally recognized research outcomes [5].

Figure 1 demonstrates the diversity and scale of publication output across Asia, with China clearly leading, followed by strong performances from India and Japan. The data reflect Asia’s growing footprint in global research, although subsequent figures and analyses are needed to assess the quality and impact of these contributions.

Table 1 provides strong evidence of Asia’s rising contribution to global research, with China leading in volume and countries like India, South Korea, and Indonesia showing significant growth. While total output varies, the upward trend across most nations reflects increasing investment in science, academic visibility, and policy-level encouragement for research dissemination. Japan demonstrates a mature output with consistency over time.

Table 2 provides a dynamic picture of how research productivity evolved across Asia. While China leads in stability and scale, countries like Indonesia and India show rapid but inconsistent growth, and others like Japan and South Korea remain steady. The data suggest a diverse and expanding research landscape, where newer players are rapidly emerging alongside established leaders. Our results also highlight the year-on-year growth in publications from Indonesia, which exhibits the highest growth rate among the top 10 Asian countries. However, its absolute publication numbers remain the lowest on average of these countries. Notably, the standard deviation (SD) for Indonesia exceeds its average publication count, indicating substantial variability in annual research output. As a result, the median value of 28 serves as a more reliable measure of central tendency for Indonesia. In contrast, other Asian countries such as China, Japan, and South Korea demonstrate stronger publication records in both volume and influence. Japan’s high H-index underscores the significant impact and enduring relevance of its research. A study by Rehman et al. concluded that Japan’s contribution to orthopaedic research is greater than that in other biomedical fields. However, the number of high-quality clinical research studies conducted in Japan within this field was meagre—comparable to other domains—highlighting the broader scope of its research influence [6].

Figure 2 captures the differential rates of research growth in Asia. While established nations like Japan show stable output, emerging contributors such as Indonesia are expanding rapidly. Meanwhile, giants like China and India continue to build momentum. The figure supports the narrative that Asia is becoming a global hub of academic productivity, though citation impact must still catch up in some nations.

Figure 3 highlights differences in research influence among Asian nations. While Japan maintains a high citation impact, countries like China and India are expanding their global footprint but still face challenges in matching citation efficiency. Smaller countries demonstrate that targeted, high-quality research can compete with larger systems in terms of impact.

Figures 4 and 5 demonstrate that citations per document is a valuable tool for assessing research efficiency and quality. While high-output countries dominate in volume, several smaller or emerging nations outperform in per-document impact**.** It underscores the importance of quality over quantity in publishing. Countries at the top of the list publish fewer documents, but each is well cited. This could indicate: high research quality or novelty, international collaboration, and/or strategic publishing in high-impact journals. Taiwan and South Korea both exhibit high CPD (citations per document) ratios, indicating that their publications are of high quality and well regarded within the academic community. A study by Nguyen et al. [7] analysed the number of articles published by East Asian countries between 1998 and 2020. Over the past two decades, there has been a strong positive trend in the total number of orthopaedic publications from China, Korea, and Japan [7].

From Fig. 6a, b, citations per document dropped sharply in 2022 for both groups, primarily because citations take time to accumulate. Notably, Asia’s citations per document (1.22) slightly exceeded the global average (1.06), suggesting growing visibility or relevance of recent Asian research. There has been an explosive growth in the number of documents produced by Asian countries (more than a 14-fold increase). While citation volume in 2022 is lower than in 1996, this is not indicative of impact decline, but rather of time-dependent citation accumulation. The steep rise in documents shows Asia’s increasing participation in global research. The slight outperformance of Asia in citations per document (2022) is encouraging and may reflect increasing international collaboration, improved research quality, or targeted publication strategies.

Figure 7 captures the early global trends of academic publishing, where a few countries led in both volume and impact. It serves as a baseline to assess how research contributions, especially from Asia, have evolved. Over time, the contrast between this figure and more recent data (e.g. Fig. 8, 2022) reveals a shift towards broader global participation in high-impact scholarly work.

While publication volume is on the rise across many countries, particularly in Asia (Fig. 9), citation performance does not always keep pace. Research quality, visibility, collaboration, and journal indexing are key to ensuring that increasing output translates into meaningful academic influence.

A rising H-index trend for Asian countries (Fig. 10) indicates growing academic influence, though some lag remains compared to Western nations. Countries with flatter or declining H-index curves may need to reassess research strategy, quality control, and visibility of outputs.

Figure 11 indicates that increased research activity leads to greater academic visibility and citation accrual. Countries aiming to improve scientific quality should balance increasing publication volume with initiatives to enhance research quality, such as peer-review rigour, funding support, and international collaboration. Those with fewer documents but high citations may reflect high-quality research or highly cited landmark papers.

The average documents for all countries in 1996 of 247.42 were associated with a large standard deviation, indicating significant variability among countries. The values for citations also were associated with high variability.

H-index typically increases over time for individual researchers or institutions as citations accumulate. However, when analysing H-index trends at a country or regional level over different publication years, several factors can cause the average H-index to appear lower in more recent years like 2022 compared to 1996. Potential reasons for the decline in H-index could be: a. citations take time to accumulate, b. fewer classic papers in recent years—highly cited papers take time to develop as given above, and c. shift towards quantity over quality—when the number of publications increases, without a corresponding increase in citations, H-index is likely to fall as is seen in cases of both Asia and all countries.

One study compared Chinese publications with those from the world’s leading publishing nations during the decade from 2005 to 2014. In that study, China had 3389 publications, compared to 31,190 from the USA and 6703 from the UK. A study by XXX and XXX reported that the total number of publications in the field of Orthopaedics and Sports Medicine from 1996 to 2022 among European countries was led by the UK (*N* = 51,510), which maintained this lead until recently, in 2022 [8]. Nearly a decade after the earlier study, China has averaged 3,000 publications annually. The total number of publications from 2013 to 2022 reached 30,088, reflecting a significant increase. Another bibliometric study by XXX et al. (2022) concluded that among 31 Asian countries, China ranked highest in total publications for 2022, followed by Japan and India. However, Singapore led in citations per document, while Japan ranked highest in H-index [9].

Notably, China has the highest percentage of self-citations at 40%, followed closely by Japan. When this metric is considered alongside the CPD (citations per document) ratio—where China ranks 22nd out of the 30 countries studied—it presents a more nuanced view of the country’s research influence. The disparity between China’s high publication volume and its relatively low CPD suggests that its research may have limited international impact and reach.

In contrast, the highest CPD values were observed in countries such as Hong Kong and Sri Lanka, indicating the substantial influence of their research on a per-document basis. These findings underscore the importance of considering multiple indicators to accurately assess the research output of different nations. Metrics such as the H-index, total citations, self-citations, and CPD provide deeper insight into the quality, impact, and longevity of research contributions.

One study analysed 30 classic articles (defined as those with TC2016 > 1000) from the Web of Science (WOS), published between 1961 and 2007 across nine countries. Among these, only Japan was listed as a co-author on one of the classic articles [10]. Research and development (R&D) has progressed significantly and is expected to include more Asian countries among global leaders.

Another study examining publications from three major Asian countries over a decade (2012–2021) concluded that, relative to population size, Japan and South Korea outperformed China in terms of publication quality. However, China led in the total number of publications among the three countries studied [11]. It is important to note that citation accumulation takes time, and the long-term impact of China’s publications may improve in the future.

A bibliometric analysis of India’s orthopaedic publications listed in the Scopus database from 2002 to 2021 revealed 4606 publications, with an average annual growth rate of 20.8% and a mean of 11.3 citations per paper. International collaboration was present in 16.3% of the publications, while only 10.4% received external research funding. The All India Institute of Medical Sciences (AIIMS), New Delhi, and the Postgraduate Institute of Medical Education and Research (PGIMER), Chandigarh, were the most prolific contributing institutions. XXX and XXX were identified as the most productive Indian authors during this period [12].

Another study examining publications from mainland China, Hong Kong, and Taiwan found that although mainland China had a high volume of publications, there remains a considerable gap in achieving the quality levels observed in Hong Kong and Taiwan [13].

A study by XXX and XXX (2024) reported substantial growth in India’s publications in orthopaedics and sports medicine in recent years. In 2022, India ranked first among South Asian Association for Regional Cooperation (SAARC) nations, third among Asian countries, and 14 th globally [14].

This bibliometric analysis may serve as a valuable tool for strategic academic planning, fostering international collaborations, and informing policy-making in orthopaedic and sports medicine research. It underscores the importance of not only increasing research output but also improving the quality and global impact of scholarly contributions.

### Limitations of our study include the following

The analysis was based on a single database search. Inclusion of additional databases could have offered a broader perspective. PubMed does not provide country-specific information in its data output; thus, we were unable to analyse this aspect. Access to more detailed data on journals, article titles, and author affiliations would have facilitated a deeper understanding of trends in research topics, journal types, and country-specific publication preferences.

Furthermore, while this study provides a comprehensive overview of publication trends, it does not explore the underlying factors contributing to the observed decline in citation counts for Asian publications. One possible explanation is the time required for citations to accumulate. On average, it takes around five years for articles to reach their peak citation count. Therefore, since the data were collected in 2022, publications from 2021 and 2022 may not have had sufficient time to fully reflect their impact through citation counts. This study did not analyse the number of publications appearing in top-ranked orthopaedic and sports medicine journals or the types of studies published (e.g. clinical trials, reviews, or observational studies). These factors can significantly influence citation counts and research impact, and their exclusion represents a limitation in fully assessing the quality of research output.

Additionally, this study focused on traditional bibliometric indicators such as total number of documents, citations, self-citations, citations per document (CPD), and H-index. Incorporating alternative metrics—such as journal impact factor, Altmetric scores, and collaboration indices—would offer a more holistic understanding of research impact.

### Recommendations

Practical steps to enhance citation impact and research quality may include encouraging greater international collaboration, encouraging regional partnerships, and investing in scientific infrastructure and funding policies. Efforts to build capacity through mentorship, training, and access to advanced research tools can further elevate the research ecosystem.

Moreover, future studies should explore the underlying challenges faced by Asian researchers, such as limited access to funding, infrastructural disparities, and language or publication barriers, all of which may hinder the visibility and influence of their work.

## Conclusions

This study revealed a remarkable surge in orthopaedic and sports medicine (OSM) publications from Asian countries, outpacing the global growth rate. While a recent decline in citation counts was observed, this likely reflects the time lag required for citations to accumulate, especially for recent publications. Nevertheless, the findings suggest a promising future for Asian research in this field.

Countries such as China and Japan are already well-established leaders in terms of research output and impact, respectively. While China has high numbers, self-citation is high at 40% and ranks low in CPD. While India’s rapid rise signals a growing reservoir of academic potential in the region, however, to translate this volume into greater global impact, there is a pressing need to improve research quality and visibility.

Metrics like the CPD, H-index, and citations are low in 2022 compared to 1996 possibly due to recency of publications. Small countries like Hong Kong, Singapore, and Sri Lanka had high CPD indicating good environment for impactful research.

The only near-significant trend is the increase in citation counts globally from 1996 to 2022. Most other comparisons—including documents and citations across Asia vs All countries—show no statistically significant differences, suggesting growing parity in publication behaviour. These findings support the view that Asia has rapidly caught up in terms of both research output and citation performance over the last two decades.

Overall, with continued support, collaboration, and strategic policy-making, Asian countries are well poised to contribute meaningfully to global orthopaedic and sports medicine research.

## Data Availability

No datasets were generated or analysed during the current study.
